# *Lactobacillus paracasei* feeding improves the control of secondary experimental meningococcal infection in flu-infected mice

**DOI:** 10.1186/s12879-018-3086-9

**Published:** 2018-04-10

**Authors:** Nouria Belkacem, Raphaëlle Bourdet-Sicard, Muhamed-Kkeir Taha

**Affiliations:** 1 Institut Pasteur, Invasive Bacterial Infections Unit , 28 rue du Dr. Roux, 75724 Paris, France; 2 Bioaster 28, rue du Docteur Roux, 75015 Paris, France; 3 Danone Research, route de la Vauve, 91120 Palaiseau, France

**Keywords:** Probiotics, Meningococci, *Neisseria meningitidis*, Influenzae, Secondary infection, Inflammation, Mice

## Abstract

**Background:**

The use of probiotics to improve anti-microbial defence, such as for influenza infections, is increasingly recommended. However, no data are available on the effect of probiotics on flu-associated secondary bacterial infections. There is strong evidence of a spatiotemporal association between influenza virus infection and invasive *Neisseria meningitidis*. We thus investigated the effect of feeding mice *Lactobacillus paracasei* CNCM I-1518 in a mouse model of sequential influenza-meningococcal infection.

**Methods:**

We intranasally infected BALB/c mice with a strain of influenza A virus (IAV) H3N2 that was first adapted to mice. Seven days later, a secondary bacterial infection was induced by intranasal administration of bioluminescent *N. meningitidis*. During the experiment, mice orally received either *L. paracasei* CNCM I-1518 or PBS as a control. The effect of *L. paracasei* administration on secondary bacterial infection by *N. meningitidis* was evaluated.

**Results:**

Oral consumption of *L. paracasei* CNCM I-1518 reduced the weight loss of infected mice and lowered the bioluminescent signal of infecting meningococci. This improvement was associated with higher recruitment of inflammatory myeloid cells, such as interstitial monocytes and dendritic cells, to the lungs.

**Conclusions:**

Our data highlight the role of the gut-lung axis*. L. paracasei* CNCM I-1518 may boost the defence against IAV infection and secondary bacterial infection, which should be further studied and validated in clinical trials.

**Electronic supplementary material:**

The online version of this article (10.1186/s12879-018-3086-9) contains supplementary material, which is available to authorized users.

## Background

Bacterial and viral respiratory infections are responsible for severe morbidity and mortality in children and adults worldwide. Among them, influenza virus is a major source of severe viral respiratory infections in adults, causing annual epidemics that result in significant morbidity and mortality. Flu pandemics during the twentieth century killed more than 100 million people [1]. Flu morbidity is often due to secondary respiratory infections by bacteria, such as *Streptococcus pneumoniae* and *Neisseria meningitidis* [2, 3]. Epidemiological studies have clearly shown a spatiotemporal association between influenza A virus (IAV) and invasive *N. meningitidis* infections [2, 4, 5]. Both infections show a seasonal pattern with most of the cases in winter. Moreover, the winter peaks of invasive meningococcal disease usually follow the peak of influenza-like syndromes [6]. This sequential pattern of flu infection and invasive meningococcal infection has been reproduced in a murine animal model [7]. The mechanisms that enhance bacterial superinfection appear to be multifactorial. Alterations in physical and immunological barriers, as well as changes in the microenvironment, have been shown to contribute to the development of secondary bacterial infections [8]. It has also been suggested that the neuraminidases (NA) of IAV enhance the adhesion process of *N. meningitidis* to the respiratory epithelium by direct interaction with the sialic-acid containing capsules that constitute a substrate for the NA of IAV. Cleavage of capsular sialic acid at the bacterial surface may unravel sub-capsular meningococcal adhesins and enhance meningococcal adhesion to epithelial cells [6].

Several clinical trials have suggested a positive effect of preventive feeding of probiotics on respiratory infections in children and the elderly [9, 10]. The administration of *Lactobacillus* species enhances protection against influenza virus infection in mice [11–14]. We have recently shown that daily consumption of *Lactobacillus paracasei* CNCM I-1518 allowed better control of IAV infection in a BALB/c mouse model, most likely through the modulation of the immune response in the respiratory system [15]. Here, we aimed to use the murine model of sequential flu-meningococcal infection to explore the effect of feeding mice *L. paracasei* CNCM I-1518 on the secondary bacterial infection.

## Methods

### Bacterial and viral strains

*Lactobacillus paracasei* strain CNCM I-1518 was cultured and prepared using identical conditions as previously described [15]. MRS (Man, Rogosa, Sharpe) broth (DIFCO laboratories Detroit, MI, USA) was used. Bacteria were harvested, washed and prepared in sterile PBS (Fisher Scientific, Graffenstaden, France) at optical density 600 nm (OD_600nm_) of 1 (Spectrometer, Biochrom, France). The bacterial suspension was stored at − 80 °C in aliquots until use.

*Neisseria meningitidis* (LNP24198lux), a bioluminescent derivative of strain 24,198 (serogroup C), was cultured on Gonococcal (GC) Base (GCB) agar medium (DIFCO laboratories Detroit, MI, USA) containing the Kellogg supplements [16]. IAV, (A/Scotland/20/74 [H3N2]), adapted to mice, was prepared, stored in aliquots at − 80 °C, and thawed prior to infection, as previously described [7].

### Mice

Six-week old female BALB/c mice were purchased (Janvier, Genest-Saint-Isle, France) and housed for one week prior to the experiment in a biosafety containment facility under the same previously described conditions [15].

### Probiotic treatment and viral and secondary bacterial infection

**Fig. 1:**
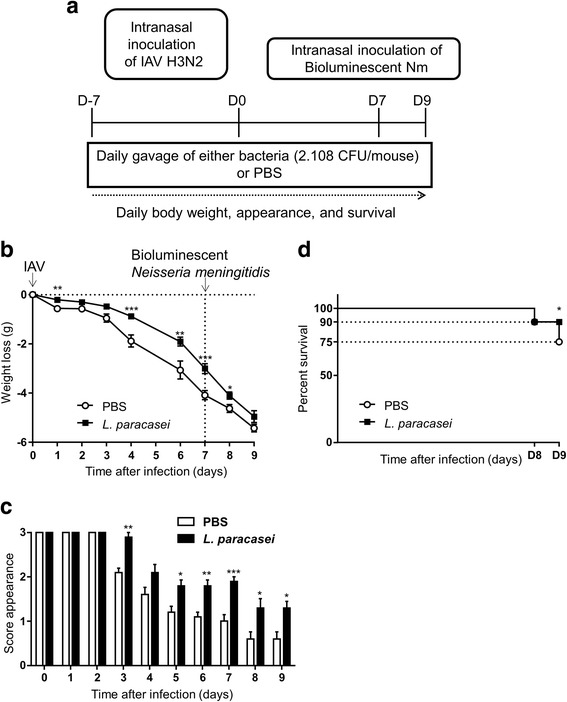
Effects of *L. paracasei* consumption on the health status of influenza/*N. meningitidis*-infected mice. **a** Schematic representation of the experimental design. **b** Body-weight loss. **c** Mortality. **d** Scores for the appearance of the mice after influenza infection as follows: 3; smooth coat, 2; patches of fur showing piloerection, 1; most of the fur showing piloerection, and 0; mouse appears “puffy”. Results are expressed as the mean ± SEM for each group (*n* = 50). (*p < 0.05, **P < 0.01, ***P < 0.001)

Three experiments were separately conducted. In each experiment, two groups of 10 and nine mice received 200 μl containing 2 × 10^8^ colony forming units (CFU)/mouse of *L. paracasei* CNCM I-1518 or 200 μl PBS (control), respectively, daily by oral gavage. Mice were infected by intranasal infection with IAV on day 0 with 50 μl H3N2 virus (260 plaque forming units, PFU) as previously described [15]. For this purpose, mice were first anesthetized by intraperitoneal injection with a mixture of xylazine and ketamine (MERIAL, Villeurbanne, France). Seven days after IAV infection (day 7), mice were superinfected in the airways with *N. meningitidis* (10^7^ CFU/ mouse in 50 μl of a bacterial suspension at 2 × 10^8^ CFU/ml). The infection was performed by the strain LNP24198lux, a serogroup C strain belonging to the hyperinvasive clonal complex ST-11 [17]. The infection was followed by dynamic imaging until 48 h after bacterial infection (Fig. 1a) as previously described [18]. Oral gavage with *L. paracasei* CNCM I-1518 was performed during the entire period of viral and secondary infection.

The health status, appearance, and survival of the mice were followed after IAV infection from day 0 to day 16 with identical scoring system as previously described [15, 19]. Survival of the mice was scored and represented as percent survival.

Mice were euthanatized at the end of experiment by injection of a high dose of a mixture of 10 mg/Kg Xylazine (Bayer, Puteaux, France) and 5 mg/Kg of Ketamine (Merial, Lyon, France). After perfusion with 3 ml PBS to wash out blood, the lungs were removed, homogenized, divided into two lots, and the homogenates used to perform the cytokine assay and flow cytometry analysis as previously described [15].

### Quantification of *N. meningitidis* (bioluminescence imaging)

Mice infected intranasally with bioluminescent *N. meningitidis* were anesthetized with a constant flow of 2.5% isoflurane mixed with oxygen, using an XGI-8 anaesthesia induction chamber (Xenogen Corp.). Bacterial infection images were acquired using an IVIS 100 system (Xenogen Corp., Alameda, CA) according to the instructions of the manufacturer. Analysis and acquisition were performed using Living Image 4.3.1 software (Xenogen Corp.). Images were acquired using a 1-min as previously described [18]. An uninfected mouse under the same conditions of acquisition was used to subtract the background.

### Determination of cytokine levels

Several cytokines (MCP1, IL10, KC, IL12p70, and IL6) were quantified in whole-lung homogenates from mice sacrificed on day 9 (48 h after *N. meningitidis* infection) in one part of the lung homogenates as mentioned above that was cleared by centrifugation at 5000 x g for 10 min at 4 °C and the supernatants used for cytokine assays using an ELISA kit (R&D Systems, Abcam®), according to the manufacturer’s instructions and the data were expressed in pg/ml of of total protein of the lungs as previously described [15].

### Antibodies and flow cytometry

The second part of whole-lung homogenates were used to prepare single-cell suspensions were using treatment with collagenase IV (Sigma-Aldrich, St. Quentin Fallavier, France) and ACK (Ammonium-Chloride-Potassium) Lysing Buffer (Fisher Scientific, Graffenstaden, France) as previously described [15, 20]. Analysis was then performed using a LSR Fortessa Flow Cytometer (BD Biosciences, San Jose, CA, USA) and the results analysed with FlowJo software (v10; Tree Star, Ashland OR, USA). The antibodies and the gating strategies used are depicted in the Additional file 1: Figure S1.

### Statistical analysis

Data are expressed as the mean s± SEM. Student’s *t* tests were used to analyse group differences. Values of *p* < 0.05 were considered to be statistically significant.

## Results

### Effect of *L. paracasei* CNCM I-1518 on the health status of mice with a secondary bacterial infection

We first assessed the effect of oral administration of *L. paracasei* CNCM I-1518 on secondary meningococcal infection. Mice were infected intranasally with H3N2 influenza virus at day 0 and superinfected intranasally with *N. meningitidis* seven days later (Fig. 1a).

After infection with influenza virus, the infected mice rapidly lost weight, but mice fed *L. paracasei* lost significantly less weight than those given PBS, as measured on days 1, 4, 6, and 7 post-infection (Fig. 1b). The mice also appeared to be ill, according to the clinical score based on the appearance of their fur. The clinical scores for the mice fed *L. paracasei* were significantly better than those of the PBS-fed mice at days 3, 4, 5, 6, and 7 post-infection (Fig. 1c). Following *N. meningitidis* infection (on day 7), the weight loss continued in both groups until day 9. However, the weight loss was less in the *L. paracasei* group and their general health was better (Fig. 1b and c). Moreover, the *L. paracasei* group showed better survival (90%) at day 9 that the PBS group (75%) (Fig. 1d). Theses data suggest that consumption of *L. paracasei* before *N. meningitidis* infection is associated with an improvement in the health of the mice.

### Effect of *L. paracasei* consumption on *N. meningitidis* respiratory infection

**Fig. 2:**
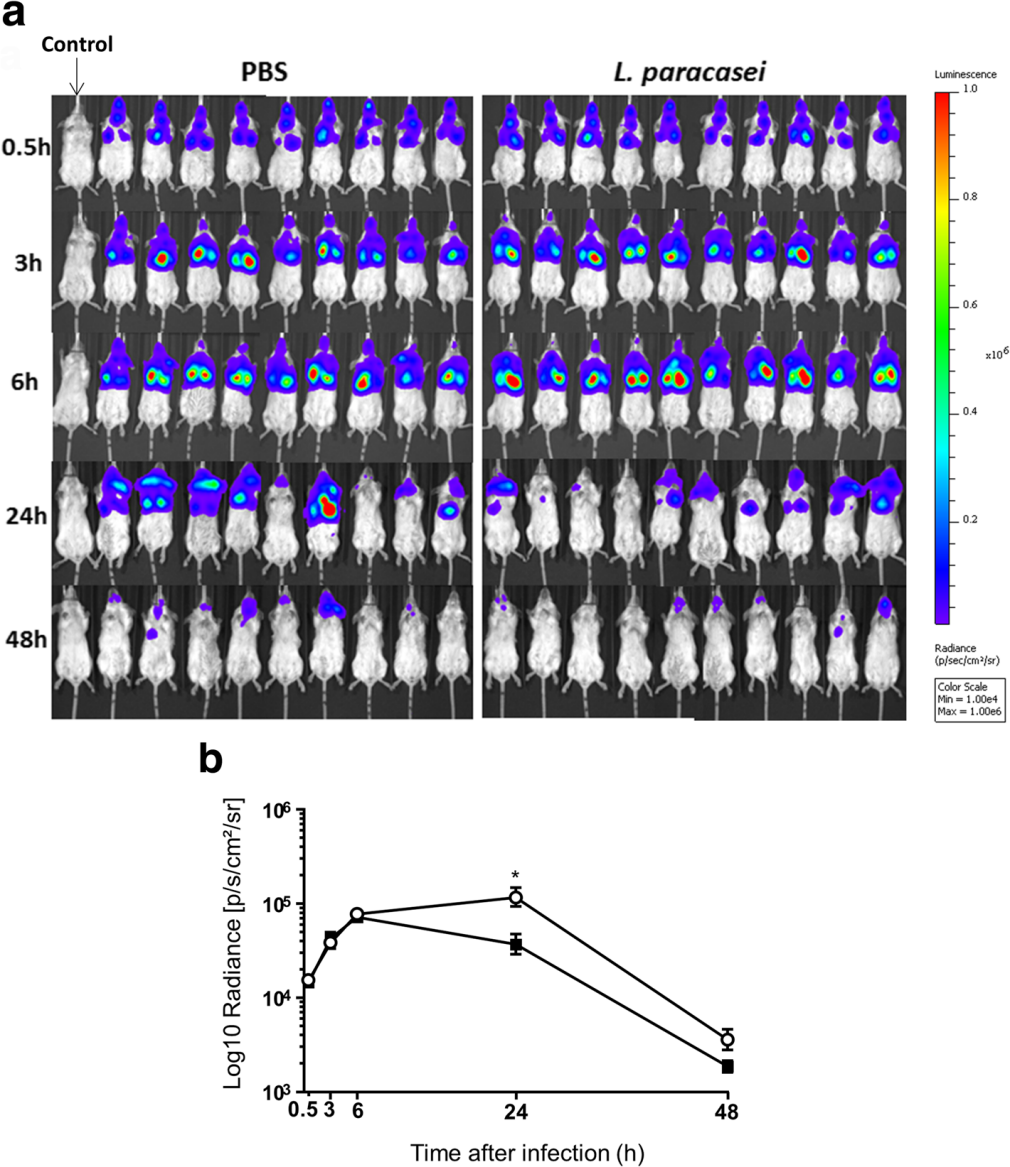
Dissemination of *N. meningitidis* in BALB/c-flu infected mice. Sequential IAV (250 PFU per mouse) and meningococcal infection (10^7^ CFU per mouse) were performed by the intranasal route. Bacterial infection was analysed by bioluminescence at the indicated times. Images depict photographs overlaid with colour representations of luminescence intensity, measured in total photons/s and indicated on the scales, in which red is the most intense and blue the least intense. **a** Ventral views of nine PBS-fed and 10 *L. paracasei*-fed mice. A non-infected mouse was added as a control. **b**. The luminescence was quantified and expressed as the means ± SEM for each category at the indicated times by defining specific representative regions of interest encompassing the entire animal

*N. meningitidis* infection was followed by dynamic bioluminescence imaging. The quantification of bacterial load was performed in IAV-infected mice for both the *L. paracasei* and PBS groups by following the total photons per second emitted by each mouse 0.5, 3, 6, 24, and 48 h after infection (Fig. 2).

Dynamic bioluminescence imaging 0.5 h after intranasal inoculation showed the bacteria to be present in the upper respiratory tract of infected mice and already starting to reach the lower respiratory pathways and lungs. The infection signal increased 3 and 6 h after *N. meningitidis* infection for both groups of mice (*L. paracasei*-fed and PBS-fed mice). The infection appeared to become established in the lower respiratory tract and lungs (Fig. 2a), with no significant difference between the two groups of infected mice. The bioluminescent signals decreased after 24 and 48 h of meningococcal infection. However, the signals were significantly lower in the *L. paracasei* fed-mice than the PBS-fed mice after 24 h of meningococcal infection. The bioluminescent signal was also lower, although not significantly, in the *L. paracasei* fed-mice than the PBS-fed mice after 48 h of infection (Fig. 2b). These data suggest gavage with *L. paracasei* was associated with faster clearance of the secondary meningococcal infection.

### Effect of *L. paracasei* consumption on cytokine profiles in the lungs

**Fig. 3:**
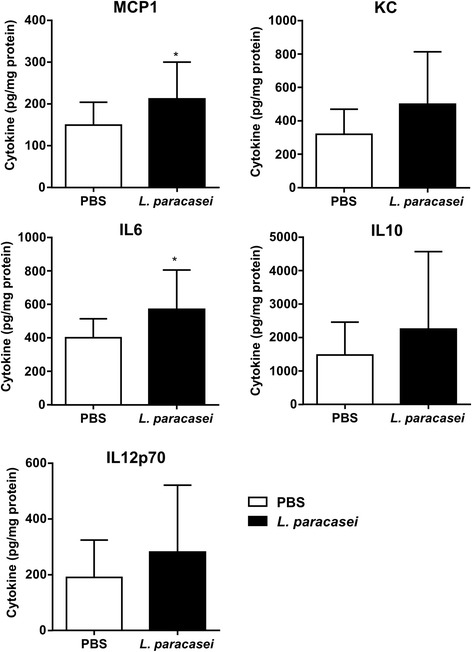
Effects of *L. paracasei* consumption on cytokine profiles relative to those of the control (PBS) group after 48 h of secondary meningococcal infection. Results are expressed as the mean ± SEM for each group (*n* = 20). Cytokines for which the differences between the two groups were significant are indicated by a star

We next investigated the effect of *L. paracasei* on the inflammatory status by quantifying the levels of several cytokines (MCP1, IL10, KC, IL12p70, and IL6) in the lungs of mice fed *L. paracasei* or PBS (Fig. 3) sacrificed on day 9 (48 h after *N. meningitidis* infection). The cytokine levels in the lungs are expressed as the amount of cytokine per mg of total protein of the tested lungs.

The levels of the inflammatory cytokines IL6, MCP1, KC, and IL12p70 were higher in the lungs of *L. paracasei*-fed than PBS-fed mice. However, only the differences in the levels of IL6 and MCP1 reached significance (Fig. 3). In addition, although the levels of the anti-inflammatory cytokine IL10 were higher in the *L. paracasei* than PBS group, the difference was not significant (Fig. 3).

### Effect of *L. paracasei* consumption on immune-cell recruitment to the lungs

**Fig. 4:**
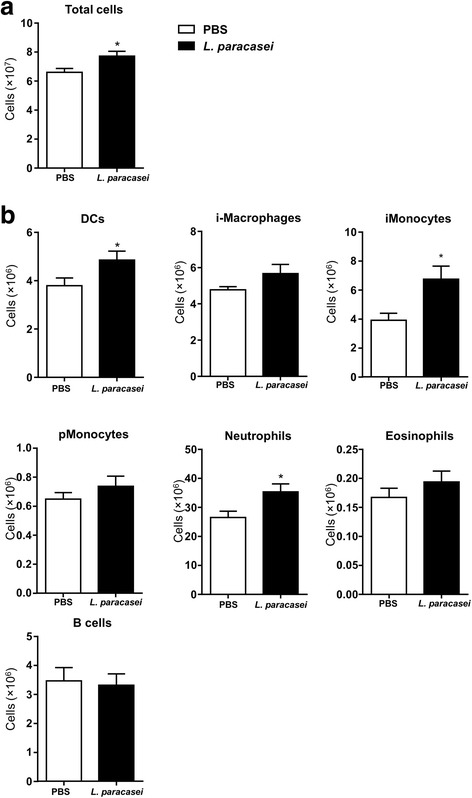
Effect of *L. paracasei* consumption on immune-cell recruitment to the lungs relative to that of the control (PBS) group after 48 h of secondary meningococcal infection. Results are expressed as the mean ± SEM for each group (*n* = 16). Cells for which the differences between the two groups were significant are indicated by a star

Innate and adaptive immune responses play a key role in controlling infection. We assessed the effect of *L. paracasei* on immune responses by quantifying total cell counts in the lungs at day 9, 48 h after *N. meningitidis* infection, using trypan blue. The cell counts in the lungs of the mice fed *L. paracasei* were significantly higher than those of the PBS-fed mice (Fig. 4a).

Flow cytometry analysis (Fig. 4b) revealed a significantly more dendritic cells, neutrophils, and inflammatory monocytes in the lungs of *L. paracasei*-fed mice sacrificed at day 9 (48 h after *N. meningitidis* infection) than those of the control group (PBS-fed mice). Although, there were more interstitial macrophages, patrolling monocytes, and eosinophils in the lungs of *L. paracasei*-fed mice sacrificed at day 9 than in control mice, the difference did not reach significance.

The flow cytometry gating strategies for immune cells are shown in the Additional file 1: Figure S1.

## Discussion

Respiratory viral infections are responsible for significant morbidity and mortality in children and adults worldwide. They also predispose individuals to secondary bacterial infections by altering the innate and adaptive immune systems [21]. Enhancing immune function through the consumption of probiotics could thus improve the outcomes of such infections [22]. We have already shown that *L. paracasei* CNCMI-1518 may exert such benefits by modulating host immune responses during IAV infection in a mouse model [15].

Here, we further show that feeding mice *L. paracasei* CNCM I-1518 not only improves the health status of mice infected with IAV, but also improves the health status of mice infected with *N. meningitis* after IAV infection. The reduction in the severity of secondary meningococcal infection may be mainly due to better control of the primary flu infection, allowing better control of the secondary bacterial infection. However, better direct clearance of *N. meningitidis* may also be possible through higher recruitment of neutrophils, inflammatory monocytes, and DCs (Fig. 4). We previously suggested that *L. paracasei* consumption induced early recruitment of immune cells, including alveolar macrophages, which may allow better control of the flu infection [15]. A role of alveolar macrophages in the protection against secondary pneumococcal infection after flu was suggested by the association between the early depletion of alveolar macrophages in the lungs and lethal pneumococcal pneumonia in flu-infected mice [23].

Indeed, we have previously reported that mice fed *L. paracasei* CNCM I-1518 prior to flu infection showed early activation of pro-inflammatory cytokines (IL-1α, IL-1β) and the massive recruitment of immune cells to the lungs (mainly DCs, macrophages, monocytes, and eosinophils) [15]. Such early recruitment may also be responsible for the faster clearance of meningococci 24 h after a secondary meningococcal infection. Moreover, we also observed differential modulation of the immune response in the lungs 48 h after secondary bacterial infection in mice fed *L. paracasei* CNCMI-1518 relative to control mice, represented by larger numbers of dendritic cells, inflammatory monocytes, and neutrophils. This may have also contributed to the control of the secondary meningococcal infection. Recent data supports a role for monocytes/macrophages in susceptibility to meningococcal sepsis in a murine model [24]. Similarly, nasal priming with viable *Corynebacterium pseudodiphtheriticum* 090104 differentially modulated the TLR3-mediated innate antiviral immune response in the respiratory tract of infant mice. This improved resistance to primary infection by the *respiratory syncytial virus* (RSV) and secondary pneumococcal pneumonia [25]. Oral administration of the probiotic *Lactococcus lactis* to mice with simultaneous vaccination with pneumococcal protective protein A was shown to reduce lung colonisation by *S. pneumoniae* [26]. Feeding mice the probiotic *Lactobacillus rhamnosus* CRL1505 (Lr05) has been reported to decrease the number of *S. pneumoniae* in the lung and prevent dissemination in the blood. This decrease was associated with increased IL6 and IL10 levels in bronchoalveolar lavages [27], consistent with our results. Mice treated with *L. rhamnosus* GG showed significantly better seven-day survival than saline-treated control mice in another experimental model of *Pseudomonas aeruginosa*-induced pneumonia [28]. This model also reveal the gut-lung axis and suggests that probiotic (*L. rhamnosus* GG) consumption may help to maintain the homeostasis of mucosal barriers [29]. Indeed, the gut microbiota has been reported to improve the outcome of experimental pneumococcal pneumonia in mice, as suggested by increased bacterial dissemination, inflammation, organ damage, and mortality in microbiota-depleted mice [30]. This beneficial effect of the gut microbiota may be due to the enhancement of primary alveolar macrophage function [30].

## Conclusions

Our data add a new facet to the impact of probiotic consumption, as they suggest a positive effect by improving recovery from a secondary bacterial infection following an influenzae infection. These observations are highly relevant as primary influenza infection enhances the effects of several types of secondary bacterial infections, such as those by *N. meningitidis*, staphylococci, pneumococci, streptococci, and *Haemophilus influenzae* [2, 31–33]. Probiotics may help to relieve the burden of such secondary bacterial infections.

## Additional file

Additional file 1: **Figure S1.** The figure describes the flow cytometry analysis of immune cells in the lungs: The used antibodies and the gating strategies. (PDF 71 kb)

## Abbreviations

CFU: colony forming unit
CNCM: Collection Nationale de Culture de Microorganismes
IAV: influenza A virus
NA: neuraminidase
PFU: plaque forming unit
