# Supplementary material for: Lactobacillus paracasei feeding improves the control of secondary experimental meningococcal infection in flu-infected mice
Source: BMC Infect Dis. 2018 Apr 10;18:167. doi: 10.1186/s12879-018-3086-9 (PMC5894232; doi:10.1186/s12879-018-3086-9)
Supplement: Supplementary file 1 — Figure S1. The figure describes the flow cytometry analysis of immune cells in the lungs: The used antibodies and the gating strategies. (PDF 71 kb) [file 12879_2018_3086_MOESM1_ESM.pdf]

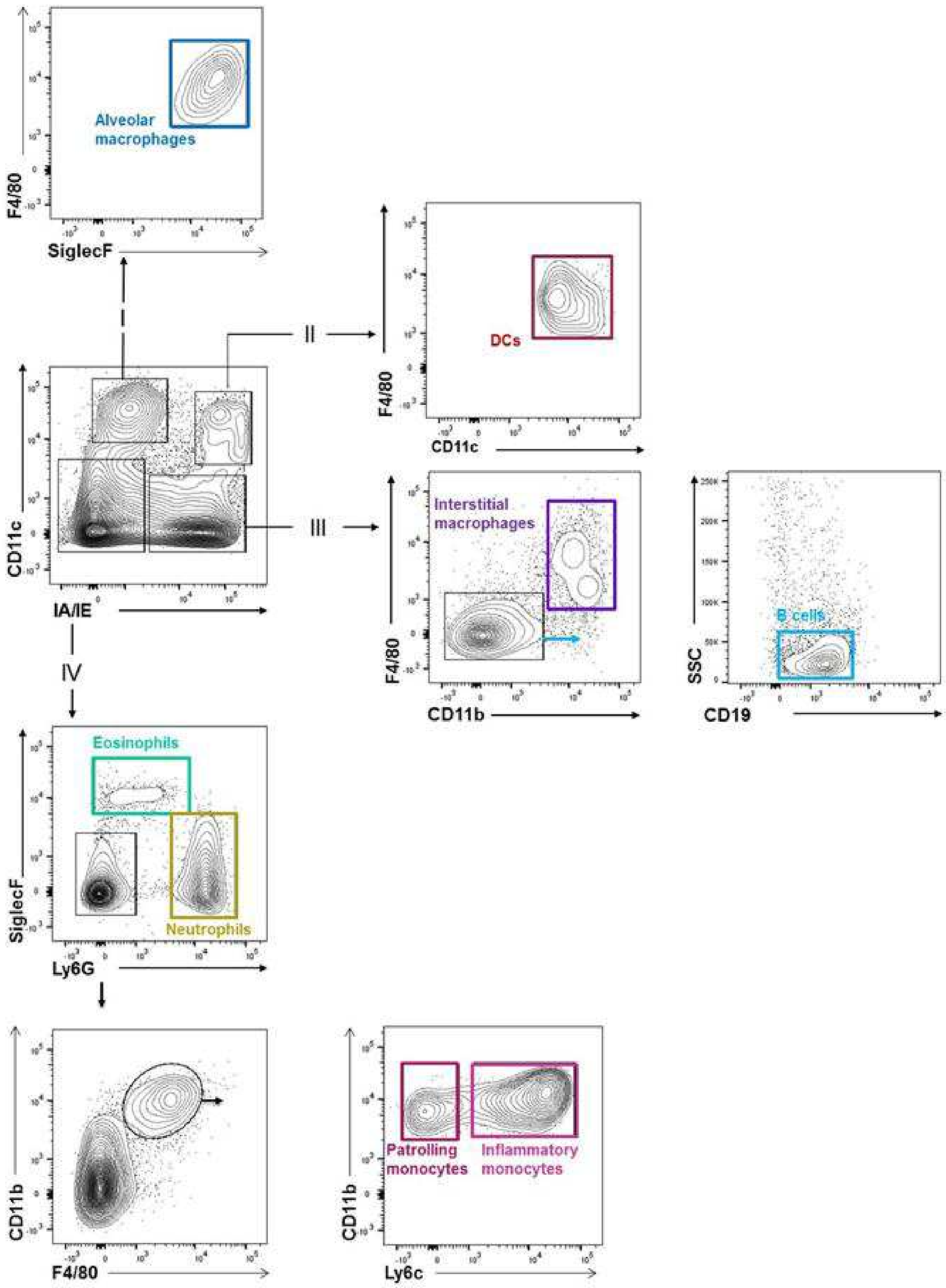

Supplementary Figure

S1Fig: Gating strategies for flow cytometry on immune cells. Alveolar macrophages were identified on the basis of the expression of IA/IE, CD11c, siglecF, F4/80. DCs were gated on the basis of high expression of CD11c, IA/IE and F4/80. Interstitial macrophages were identified from CD11c-, IA/IE+ cells on the basis of their expression of both CD11b and F4/80 expression. B cells were identified on the basis of their expression of IA/IE and CD19 after gating out interstitial macrophages. CD11c^{low}, IA/IE- are composed of eosinophils, neutrophils and monocytes that were gated as follow: Eosinophils were identified on the basis of their high expression of siglecF and intermediate expression of Ly6G. Neutrophils were identified on the basis of their expression of Ly6G. Monocytes were identified as F4/80 intermediate on Ly6G- and SiglecF- after gating out neutrophils and eosinophils. Monocytes were then separated into inflammatory monocytes Ly6c+ and patrolling monocytes CD11b+
